# Reversible cerebral vasoconstriction syndrome with delayed vasospasms and subdural hematoma after cardiac transplantation: a case report

**DOI:** 10.3389/fcvm.2025.1660432

**Published:** 2025-10-28

**Authors:** Shuhei Egashira, Manabu Inoue, Tasuku Hada, Satsuki Fukushima, Yasumasa Tsukamoto, Masatoshi Koga, Kazunori Toyoda

**Affiliations:** ^1^ Department of Cerebrovascular Medicine, National Cerebral and Cardiovascular Center, Suita, Japan; ^2^ Department of Transplant Medicine, National Cerebral and Cardiovascular Center, Suita, Japan; ^3^ Division of Cardiac Surgery, National Cerebral and Cardiovascular Center, Suita, Japan

**Keywords:** reversible cerebral vasoconstriction syndrome, cardiac transplantation, intracranial hemorrhage, ischemic stroke, subdural hematoma

## Abstract

**Background:**

Reversible cerebral vasoconstriction syndrome (RCVS) is a rare but severe neurovascular complication following cardiac transplantation. Its diagnosis is often complicated by atypical presentations and by neuroimaging limitations, as transplant-related devices can hinder timely magnetic resonance (MR) imaging.

**Case presentation:**

A 59-year-old woman developed a severe headache 9 days post-cardiac transplantation. Neurological examination revealed left lower quadrantanopia. A head Computed Tomography (CT) scan showed a subdural hematoma, along with a subcortical and convexity subarachnoid hemorrhage in the right parieto-occipital lobe. Initial CT angiography showed only focal arterial stenosis. On day 10, she developed left hemineglect. After removal of transplant-related devices, MR imaging showed a watershed infarct and MR angiography revealed multiple vasospasms.

**Diagnostic challenges:**

The diagnostic challenge was the delayed onset of diffuse vasospasms, the confirmation of which was precluded by the initial contraindication to MR imaging.

**Management:**

The patient was diagnosed with RCVS. Management included withdrawal of tacrolimus and initiation of oral verapamil and prasugrel. This led to rapid clinical and radiological improvement.

**Conclusion:**

This case highlights that RCVS can present with a subdural hematoma before the onset of delayed vasospasm after cardiac transplantation. Repeated cerebrovascular evaluations are crucial for timely diagnosis and management in post-transplant patients with unexplained headaches or intracranial hemorrhage.

## Introduction

Reversible cerebral vasoconstriction syndrome (RCVS), a cerebrovascular syndrome characterized by severe headaches and reversible constriction of cerebral arteries, has been reported as a rare complication of cardiac transplantation (1, 2). The etiology of this condition is recognized as multifactorial, often resulting from a convergence of factors including calcineurin inhibitor (CNI) toxicity, labile hypertension, and the physiological stress of surgery (1). These triggers overlap with those of posterior reversible encephalopathy syndrome (PRES), and the two disorders can occasionally coexist (2, 3). The diagnosis of RCVS is inherently complex, as patients may present with atypical hemorrhagic or ischemic strokes, and the typical multiple vasospasms are frequently not apparent in the early stages of the disease (4). This problem is further complicated after cardiac transplantation by devices that hinder timely magnetic resonance (MR) imaging. We describe a case of RCVS presenting with a subdural hematoma before the onset of delayed vasospasm after cardiac transplantation, adding to the literature by highlighting this atypical presentation and its associated diagnostic challenges.

## Case description

**Figure 1:**
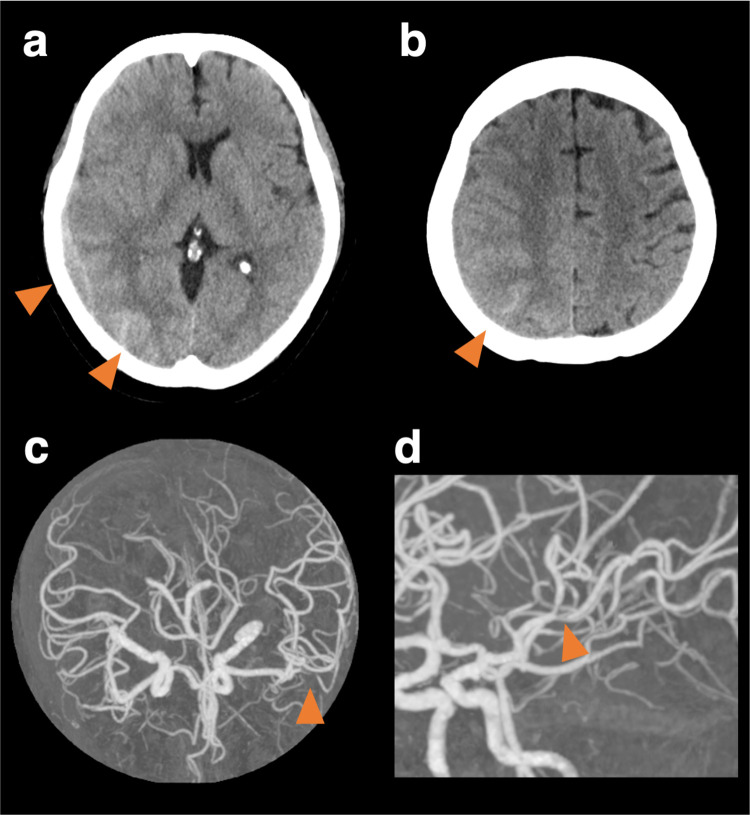
Cranial computed tomography and computed tomography angiography at the onset. Cranial computed tomography (CT) shows subcortical hemorrhage in the right posterior lobe, subdural, and convexity subarachnoid hemorrhage **(a, b)**. CT angiography shows vasoconstriction limited to the left middle cerebral artery, though it was unclear whether it was a vasospasm or mere stenosis **(c, d)**.

**Figure 2:**
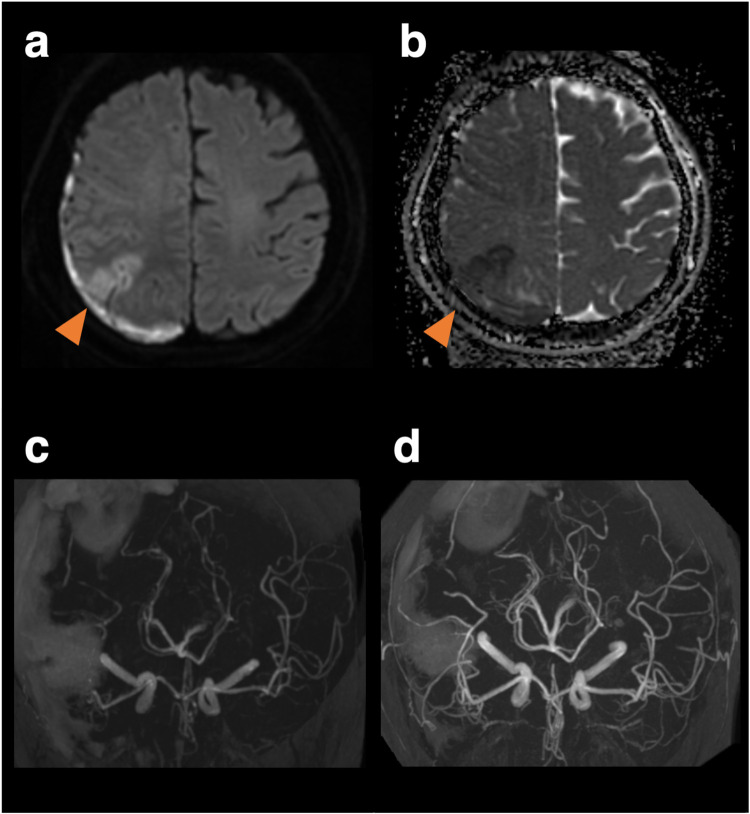
Magnetic resonance images and angiography on the 10th day when the patient developed left hemineglect, and magnetic resonance angiography on the 18th day of follow-up. Diffusion-weighted imaging **(a)** and apparent diffusion coefficient map **(b)** reveals a watershed infarct. Vasoconstriction peaked on day 10 **(c)** and improved on day 18 **(d).**

A 59-year-old woman with cardiomyopathy due to Duchenne muscular dystrophy underwent cardiac transplantation. Her pre-transplant history included muscular dystrophy-related weakness but no history of hypertension, migraine, or other significant cardiovascular comorbidities. Her perioperative immunosuppression regimen consisted of tacrolimus, mycophenolate mofetil, and prednisolone. She had been taking aspirin for donor-transmitted coronary atherosclerosis. Her postoperative course was complicated by labile hypertension, with systolic pressures ranging from 140 to 180 mmHg. On day nine, she developed a headache. Neurological examination revealed left lower quadrantanopia. A head Computed Tomography (CT) scan showed a subdural hematoma with a maximum thickness of 1 cm, along with a subcortical and convexity subarachnoid hemorrhage in the right parieto-occipital lobe (Figures 1a,b). CT angiography revealed stenosis confined to the left middle cerebral artery, though it was unclear whether this represented vasospasm or mere stenosis (Figures 1c,d). MR imaging was not immediately available due to the presence of transplant-related devices. Discontinuation of aspirin did not prevent the development of left hemineglect on the 10th day. After the transplant-related devices were removed, cranial MR images on the 10th day revealed a watershed infarct in the right parietal lobe (Figures 2a,b), and MR angiography demonstrated diffuse, multifocal moderate vasospasms affecting the bilateral anterior, middle, and posterior cerebral arteries (Figure 2c). She was diagnosed with RCVS. Management included immediate withdrawal of tacrolimus and initiation of oral verapamil. Following the identification of the watershed infarct, an antiplatelet agent was considered for secondary stroke prevention. Prasugrel was chosen over other P2Y12 inhibitors because its antiplatelet effect is not influenced by CYP2C19 loss-of-function variants, which are prevalent in Asian populations (5). This regimen led to an improvement in her neurologic symptoms within 24 h, and follow-up MR angiography on day 18 showed improvement in the vasospasm (Figure 2d). Her immunosuppressive regimen was adjusted to a combination of prednisolone, everolimus, and mycophenolate mofetil, and then gradually tapered. She was discharged home on day 49. At a 6-month follow-up visit, she remained neurologically stable with a persistent, isolated left lower quadrantanopia but was independent in her activities of daily living (modified Rankin Scale score of 1). Follow-up cranial magnetic resonance imaging at 6 months revealed no new infarction or hemorrhage and no recurrence of vasospasm.

## Discussion

We reported a case of RCVS with a subdural hematoma before the onset of delayed vasospasm after cardiac transplantation. While RCVS is a known, albeit rare, complication post-transplantation, our case is distinguished by its initial presentation with a subdural hematoma, which is an uncommon manifestation of RCVS. Furthermore, the delayed onset of diffuse, multifocal vasospasms, which were not apparent on initial angiogram, highlights a diagnostic pitfall. This clinical course, combined with the diagnostic challenge posed by contraindications to immediate MR imaging due to transplant-related devices, adds a critical perspective to the existing literature on post-transplant neurovascular complications.

The pathophysiology of RCVS in the post-transplant setting is complex. Since tacrolimus is a known CNI trigger, the rapid clinical improvement following its withdrawal suggests it played a key role; however, this interpretation should be made with caution. The improvement may also be partly attributed to the natural, self-limiting course of RCVS or the therapeutic effect of the calcium channel blocker that was initiated. Therefore, it is most plausible that the clinical course was influenced by a convergence of these factors. Although specific biomarkers such as serum tacrolimus, endothelin-1, or catecholamine levels were not measured in our patient, CNI-induced endothelial dysfunction is a well-described mechanism, thought to involve an imbalance of vasoactive substances like reduced nitric oxide and increased endothelin-1 (6). The additional physiological stress from surgery and altered catecholamine dynamics from the denervated heart likely contributed to a vulnerable cerebrovascular state in this patient (7).

The clinical course of RCVS is varied, typically starting with a sudden, severe headache. While ischemic and hemorrhagic complications are common, subdural hematomas are a rarer manifestation (2%–4%) (8–11). Cerebral infarction often occurs days after the initial headache or hemorrhage, frequently affecting watershed zones, as seen in our patient (10). Vasospasm typically begins distally, and the characteristic multifocal involvement of major vessels may not be apparent on initial imaging, with vasospasm peaking on average around 16 days after onset (4).

Treatment for RCVS focuses on removing the offending agent and initiating calcium channel blocker. Given that CNIs are often essential for preventing graft rejection, a switch to an alternative agent, such as the mTOR inhibitor everolimus, is one of the reasonable strategies.

This case provides new information that could modify existing clinical practice by demonstrating that an initial angiogram does not rule out evolving RCVS in post-transplant patients. The primary implication for clinicians is that a low threshold for repeat vascular imaging, preferably within 3–7 days, is warranted for post-transplant patients who present with thunderclap headache, focal deficits, or intracranial hemorrhage. This proactive approach is particularly crucial for patients who exhibit an atypical clinical course, such as the co-occurrence of subdural hematoma and cerebral infarction seen in our case, even if initial findings are non-diagnostic.

### Limitations

This study has several limitations. First, as a single case report, the findings cannot be generalized. Second, specific biomarkers that could have provided further insight into the pathophysiology, such as serum tacrolimus levels, endothelin-1, or catecholamines, were not measured. Third, the diagnosis and follow-up of vasospasm were based on MR angiography; digital subtraction angiography was not performed.

### Future directions

This case underscores the need for further research to better define the incidence, risk factors, and optimal management strategies for RCVS in patients after cardiac transplantation. Future research could also focus on identifying non-invasive biomarkers to facilitate earlier diagnosis and on establishing standardized protocols for cerebrovascular monitoring in this high-risk population.

## Patient perspective

After my heart transplant, just as I was starting to recover, I suddenly experienced a severe headache and visual disturbances, which made me feel very anxious. At first, not knowing the cause was frightening, but thanks to thorough testing and clear explanations, I was able to receive an accurate diagnosis and appropriate treatment. I hope my experience can offer encouragement to others who may face health issues after transplantation.

## Data Availability

The original contributions presented in the study are included in the article/Supplementary Material, further inquiries can be directed to the corresponding author.
